# Surgical treatment and reproductive outcomes in caesarean scar pregnancy at a single center

**DOI:** 10.1186/s12958-024-01225-7

**Published:** 2024-05-11

**Authors:** Yan Lei, Xin Du, Yu Liu, Fangshu Le, Jianshan Zhou

**Affiliations:** Department of Gynecology, Maternal and Child Health Hospital of Hubei Province, Tongji Medical College, Huazhong University of Science and Technology, Wuhan, 430070 China

**Keywords:** Caesarean scar pregnancy, Reproductive outcomes, Ultrasound-guided uterine aspiration, Laparoscopic scar repair

## Abstract

**Background:**

To investigate factors associated with different reproductive outcomes in patients with Caesarean scar pregnancies (CSPs).

**Methods:**

Between May 2017 and July 2022, 549 patients underwent ultrasound-guided uterine aspiration and laparoscopic scar repair at the Gynaecology Department of Hubei Maternal and Child Health Hospital. Ultrasound-guided uterine aspiration was performed in patients with type I and II CSPs, and laparoscopic scar repair was performed in patients with type III CSP. The reproductive outcomes of 100 patients with fertility needs were followed up and compared between the groups.

**Results:**

Of 100 patients, 43% had live births (43/100), 19% had abortions (19/100), 38% had secondary infertility (38/100), 15% had recurrent CSPs (RCSPs) (15/100). The reproductive outcomes of patients with CSPs after surgical treatment were not correlated with age, body mass index, time of gestation, yields, abortions, Caesarean sections, length of hospital stay, weeks of menopause during treatment, maximum diameter of the gestational sac, thickness of the remaining muscle layer of the uterine scar, type of CSP, surgical method, uterine artery embolisation during treatment, major bleeding, or presence of uterine adhesions after surgery. Abortion after treatment was the only risk factor affecting RCSPs (odds ratio 11.25, 95% confidence interval, 3.302–38.325; *P* < 0.01) and it had a certain predictive value for RCSP occurrence (area under the curve, 0.741).

**Conclusions:**

The recurrence probability of CSPs was low, and women with childbearing intentions after CSPs should be encouraged to become pregnant again. Abortion after CSP is a risk factor for RCSP. No significant difference in reproductive outcomes was observed between the patients who underwent ultrasound-guided uterine aspiration and those who underwent laparoscopic scar repair for CSP.

## Background

Caesarean scar pregnancy (CSP), in which a pregnancy sac is implanted at the site of a previous incisional scar [1], is one of the most serious complications of Caesarean sections (CSs), with an incidence of 1:216–1:1800, in 1.15% of women with a history of CSs [2, 3]. If CSP is detected in the first trimester, it may cause massive uncontrollable bleeding during and after uterine aspiration, uterine rupture, damage to surrounding organs, and hysterectomy in severe cases [4]. Placental accretion and even dangerous placenta previa may occur in the middle and third trimesters of CSP [5]. Surgical treatment is the preferred method for the treatment of CSP [3]. However, to date, the relationship between surgical treatment modalities for CSP and reproductive outcomes has not been fully evaluated because of the diversity and uncertainty of CSP treatment modalities owing to different types of CSP, economic constraints, and physicians’ personal experiences and habits [6, 7]. Petersen et al. summarised the 14 main treatment methods for CSP and recommended the following surgical methods: uterine aspiration, laparoscopic surgery, hysteroscopy, and vaginal surgery [8]. In this study, ultrasound-guided uterine aspiration and laparoscopic scar repair were selected as the surgical treatment methods for CSP.

The influence of different surgical methods on the reproductive outcomes has been debated. Some reports have suggested that laparoscopic repair of scar defects can effectively improve fertility and clinical pregnancy rates [9–11]. However, some scholars are sceptical of this conclusion, believing that the sample size and follow-up of these studies were insufficient to analyse fertility or reproductive outcomes [12]. A multivariate analysis has demonstrated that the intrauterine pregnancy rates in the groups treated with surgery without Caesarean scar resection and the group treated with surgery for Caesarean scar resection were 80.1% and 86.0%, respectively, without significant differences in reproductive outcomes [13]. A recent review has also indicated that uterine scar repair surgery did not significantly affect the reproductive outcomes of patients with CSP [14].

Hence, we designed a retrospective study and followed up 549 patients after CSP treatment for 5 years to understand their reproductive intentions and track their reproductive outcomes, aiming to provide evidence to further clarify whether different surgical treatments have an impact on reproductive outcomes. We also explored the possible factors influencing different reproductive outcomes in patients with CSP after treatment to provide suggestions, help clinicians select CSP treatment plans, and guide patients with CSP to become pregnant again.

## Methods

### Patients

**Fig. 1:**
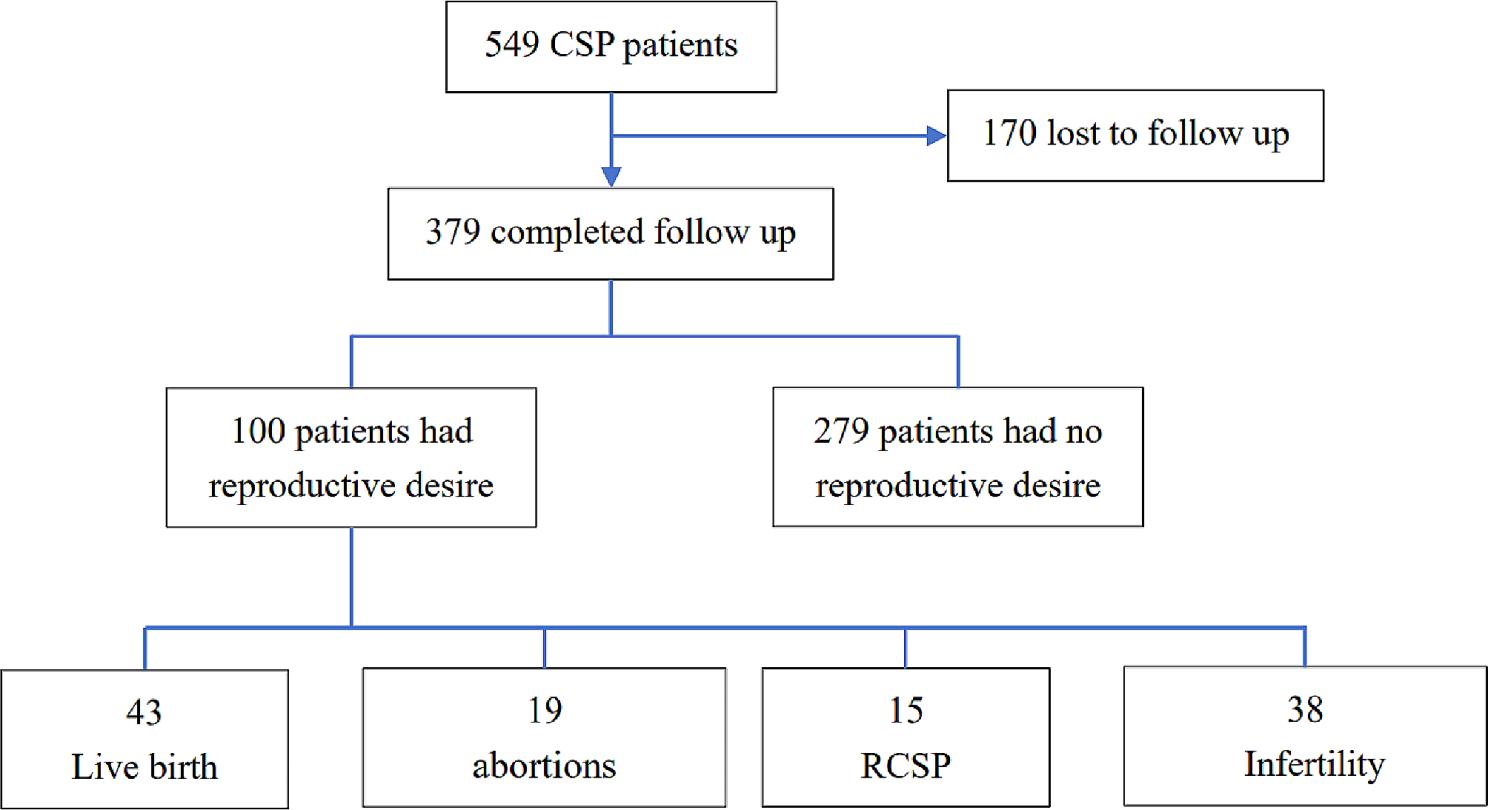
Outcomes of subsequent pregnancies. CSP, cesarean scar pregnancy.RCSP, recurrent cesarean scar pregnancy

By 30 October 2023 549 patients with CSP who were admitted to the Gynaecology Department of Hubei Maternal and Child Health Hospital between May 2017 and July 2022 were interviewed by telephone. After excluding patients not desiring fertility and those lost to follow-up, 100 patients who still desired to conceive again after the initial treatment were included in the study, and their clinical data were collected. The inclusion criteria were patients with CSP and definite imaging diagnoses and patients who underwent uterine aspiration or laparoscopic excision of the deficient uterine scar with a gestational sac (GS), followed by uterine scar defect repair. The exclusion criteria were as follows: patients undergoing expectant treatment, hysteroscopies, vaginal surgeries, and drug treatment for CSP; patients without desire for fertility and those who were lost to follow-up; and patients with congenital uterine malformations. The final reproductive outcomes of the 100 patients included live births, abortions, recurrent CSP (RCSP), and secondary infertility. A flowchart of the study is shown in Fig. 1.

This study was approved by the Ethics Committee of Hubei Maternal and Child Health Hospital (registration number: 2023IEC123), and patient informed consent was obtained via telephone follow-up.

### Reproductive outcomes and grouping methods

In total, 100 patients were grouped and compared according to four types of reproductive outcomes: the patients were divided into the live birth and non-live birth groups according to whether they eventually obtained a live birth; the patients were divided into abortion and non-abortion groups according to whether they had an abortion (including spontaneous and missed abortions); and the patients were divided into RCSP and non-RCSP groups according to whether they had CSP again. The interval between the end of initial treatment and telephone follow-up was approximately 1–5 years. Patients who never became pregnant during this period were included in the secondary infertility group, and the remaining patients were included in the non-secondary infertility group.

### Diagnostic criteria

CSP was diagnosed using the following ultrasonographic diagnostic criteria: (1) no GS was detected in the uterine cavity or cervical canal; (2) the placenta or GS was implanted in the uterine scar; (3) triangular (≤ 8 weeks) or round/oval (> 8 weeks) GSs filled with cicatricial “diverticulum” was observed; (4) the muscular layer between the GS and bladder became thinner (1–3 mm) or disappeared; (5) rich or significant blood flow signals were observed in the CS scar; and (6) the embryo and/or yolk sac was visible with or without cardiac tube pulsations. CSP is classified into types I, II, and III based on the relationship between the position of GS and myometrial thickness (MT) on ultrasonography.CSP with MT > 3 mm and ≤ 3 mm was defined as type I and type II, respectively. In typeI and II CSP, the GS was partially implanted in the scar or cavity. CSP with MT ≤ 3 mm was defined as type III, and the GS of type III protrudes from the CS scar or forms an amorphous mass with rich vascularity at the CS scar [15]. Ultrasound scans were performed by one of multiple sonographers in training who had either performed > 3,000 scans or had > 1 year of experience. Each woman was examined by a sonographer who was blinded to the other measurements and was included only once in the study.

### Surgical methods

The surgical methods used were either ultrasound-guided uterine aspiration or laparoscopic pregnancy removal and scar repair. According to the Expert Opinion of Diagnosis and Treatment of Caesarean Scar Pregnancy (2016) [15], patients with type I CSP with stable vital signs and a gestational age of < 8 weeks were selected. Patients with type I and II CSPs with a gestational age of ≥ 8 weeks were selected for ultrasound-guided uterine aspiration before uterine artery embolisation. Type III CSP is characterised by a thin muscle layer at the scar of the anterior uterine wall and is treated with laparoscopic pregnancy removal and scar repair.

### Statistical analysis

SPSS (version 22.0) was used for the data analysis, and statistical significance was set at *P* < 0.05. Count data with normal distributions are described as frequencies (%), and non-parametric data from the Mann–Whitney U test for comparing between groups are presented as medians (interquartile ranges). Pearson’s chi-square or Fisher’s exact probability tests were used to compare the two groups. Differences between the two groups were compared using a two-independent sample t-test. An exploratory stratified analysis was performed, and the influencing factors were adjusted using a logistic regression model. An exploratory stratified analysis was performed, and the influencing factors were adjusted using a logistic regression model. Receiver operating characteristic (ROC) curves were plotted to evaluate the predictive value of the factors influencing reproductive prognosis, and the area under the curve (AUC) was calculated. AUC values of > 0.5 indicated predictive capability, and AUC values of > 0.7 indicated a certain accuracy of prediction.

## Results

### Reproductive outcomes after CSP treatment

Among the 549 patients with CSP, 170 were lost to follow-up, 279 patients (279/379, 73.6%) withdrew their intention to continue their pregnancy, 100 patients (100/379, 26.4%) still had fertility requirements, 43 (43/100, 43%) had live births, 19 (19/100, 19%) had abortions, 38 (38/100, 38%) had infertility, and 15 (15/100, 15%) had RCSP. Among the 43 live births, 32 (32/43, 74.4%) were full term, 11 (11/43, 25.6%) were preterm, 1 (1/43, 2.3%) was preterm with placental implantation and massive bleeding, and the other 10 were preterm for reasons unrelated to placental implantation disease. Eleven (11/38, 28.9%) intrauterine adhesions (IUAs) occurred in the non-pregnant group.

### Analysis of influencing factors in patients with CSP with live birth outcomes

The univariate analysis revealed that patients in the live birth group had a fewer frequency of pregnancy (median 4.0 vs. 5.0) and abortion (median 2.0 vs. 2.2) than the non-live birth group. The incidences of IUA (0% vs. 19.3%), abortion after treatment (0% vs. 33.3%), secondary infertility (0% vs. 66.7%), and RCSP (4.7% vs. 22.8%) were significantly lower in the in the live birth group than in the non-live birth group (*P* < 0.05, Table 1). No significant differences in age, length of stay, body mass index (BMI), number of pregnancies, abortions, CSs before treatment, number of weeks of menopause during treatment, maximum GS diameter, thickness of the remaining muscle layer of the uterine scar, type of CSP, method of treatment, uterine artery embolisation (UAE), major bleeding, or whether persistent Caesarean scar pregnancy after treatment were observed between the two groups (*P* > 0.05, Table 1). The results of the univariate analysis were included in the logistic multifactorial analysis, which showed that none of the factors affected the reproductive outcomes of live births after CSP.

**Table 1:** Comparison of clinical data of pregnancy outcome between live birth group and non-live birth group

Clinical Coefficients	Live birth group (*n* = 43)	Non-live birth group (*n* = 57)	t / χ2 /z	*P*
Age [years, M (P-25P75)]	30.0 (29.0–35.0)	32 (30.0–36.0)	1.398	0.162
Length of stay [days, M (P25-P75)]	5.0 (4.0–6.0)	5.0 (4.0–6.0)	0.746	0.445
Pregnancy [times, M (P25-P75)]	4.0 (3.0–5.0)	5.0 (3.0–6.0)	2.858	0.004
Yield [times, M (P25-P75)]	1.0 (1.0–1.0)	1.0 (1.0–1.0)	1.458	0.145
Cesarean section[times, M (P25-P75)]	1.0 (1.0–1.0)	1.0 (1.0–1.0)	1.957	0.05
Abortion [times, M (P25-P75)]	2.0 (1.0–3.0)	2.2 (1.0–4.0)	2.408	0.016
Weeks of menopause [days, M (P25-P75)]	6.60 (6.20–7.50)	7.15 (6.30–8.85)	1.285	0.199
Gestational sac diameter [cm, M(P25-P75)]	2.60 (2.00-3.50)	2.90 (2.05–4.60)	1.473	0.141
MT [cm, M (P25-P75)]	0.31 (0.20–0.43)	0.27 (0.20–0.40)	0.571	0.568
BMI [kg/m2, *M* (P25-P75)]	21.61 (20.20-24.61)	21.30 (19.34–22.85)	1.452	0.147
Method of surgery				
Uterine aspiration [n(%)]	86.0% (37/43)	73.7% (42/57)		
Laparoscopic surgery [n(%)]	14.0% (6/43)	26.3% (15/57)	2.284	0.319
UAE [example (%)]	16.3% (7/43)	19.3% (11/57)	0.151	0.796
Major bleeding [n(%)]	0% (0/43)	3.5% (2/57)	1.540	0.505
Abortion after treatment [n(%)]	0% (0/43)	33.3% (19/57)	17.695	< 0.01
Secondary infertility [n (%)]	0% (0/43)	66.7% (38/57)	46.237	< 0.01
IUA [n(%)]	0% (0/43)	19.3% (11/57)	9.324	0.002
PCSP [n(%)]	9.3% (4/43)	7.0% (4/57)	0.174	0.722
RCSP [n(%)]	4.7% (2/43)	22.8% (13/57)	6.337	0.021
Type of CSP				
I [n(%)]	53.5 (23/43)	42.1% (24/57)		
II [n(%)]	39.5% (17/43)	43.9% (25/57)	1.895	0.388
III [n(%)]	7.0% (3/43)	14.0% (8/57)		

CSP: Caesarean scar pregnancy; RCSP: Recurrent caesarean scar pregnancy; UAE: Artery embolization; MT: Myometrial thickness; IUA: intrauterine adhesions; PCSP: persistent caesarean scar pregnancy

### Analysis of influencing factors in patients with CSP with abortion outcomes after treatment

In the univariate analysis, patients in the abortion group had significantly more pregnancies (median 5.0 vs. 4.0), lower BMIs (median 20.0 vs. 21.6), lower incidences of live birth (0% vs. 53.1%) and secondary infertility rates (0% vs. 46.9%), and higher RCSP rates (47.4% vs. 7.4%) than the non-abortion group (*P* < 0.05, Table 2). Meanwhile, no other indicators were statistically significant (*P* > 0.05; Table 2). The results of the univariate analysis were incorporated into the logistic multivariate analysis, which showed that these factors did not affect the reproductive outcomes of abortion after CSP.

**Table 2:** Comparison of clinical data for pregnancy outcomes in abortion and non-abortion groups

Clinical Coefficients	Abortion group (*n* = 19)	Non-abortion group (*n* = 81)	t / χ2 /z	*P*
Age [years, M (P-25P75)]	31.0 (28.0–33.0)	32.0 (30.0–36.0)	1.932	0.053
Days in hospital [days, M (P25-P75)]	5.0 (4.0–5.0)	5.0 (4.0–6.0)	0.401	0.688
Pregnancy[times, M (P25-P75)]	5.0 (3.0–6.0)	4.0 (3.0–5.0)	2.075	0.038
Yield [times, M (P25-P75)]	1.0 (1.0–1.0)	1.0 (1.0–1.0)	0.475	0.635
Cesarean section[times, M (P25-P75)]	1.0 (1.0–1.0)	1.0 (1.0–1.0)	0.894	0.371
Abortion [times, M (P25-P75)]	3.0 (1.0–4.0)	2.0 (1.0–3.0)	1.915	0.055
Weeks of menopause [days, M (P25-P75)]	6.70 (6.08–8.25)	7.10 (6.28–8.33)	0.758	0.448
Gestational sac diameter [cm, M(P25-P75)]	2.80 (1.80–3.90)	2.70 (2.05–3.95)	1.473	0.141
MT[cm, M (P25-P75)]	0.32 (0.23–0.40)	0.28 (0.20–0.40)	0.259	0.795
BMI [kg/m2, *M* (P25-P75)]	20.00 (18.90 -21.87)	21.61 (20.20-24.19)	2.478	0.013
Method of surgery				
Aterine aspiration [n(%)]	84.2% (16/19)	77.8% (63/81)		
Laparoscopic surgery [n(%)]	15.8% (3/19)	22.2% (18/81)	0.752	0.686
UAE [n(%)]	15.8% (3/19)	18.5% (15/81)	0.078	0.780
Major bleeding [n(%)]	0% (0/19)	2.5% (2/81)	0.479	0.489
Live birth [n(%)]	0% (0/19)	53.1% (43/81)	17.695	< 0.01
RCSP [n(%)]	47.4% (9/19)	7.4% (6/81)	19.275	< 0.01
Secondary infertility[n (%)]	0% (0/19)	46.9% (38/81)	14.377	< 0.01
IUA [n (%)]	5.3% (1/19)	12.3% (10/81)	0.789	0.375
PCSP [n(%)]	5.3% (1/19)	8.6% (7/81)	0.239	0.625
Type of CSP				
I [n(%)]	57.9% (11/19)	44.4% (36/81)		
II [n(%)]	36.8% (7/19)	43.2% (35/81)	1.443	0.486
III[n(%)]	5.3% (1/19)	12.4% (10/81)		

CSP: Caesarean scar pregnancy; RCSP: Recurrent caesarean scar pregnancy; UAE: Artery embolization; MT: Myometrial thickness; IUA: intrauterine adhesions; PCSP: persistent caesarean scar pregnancy

### Analysis of influencing factors in patients with CSP with RCSP outcomes

**Fig. 2:**
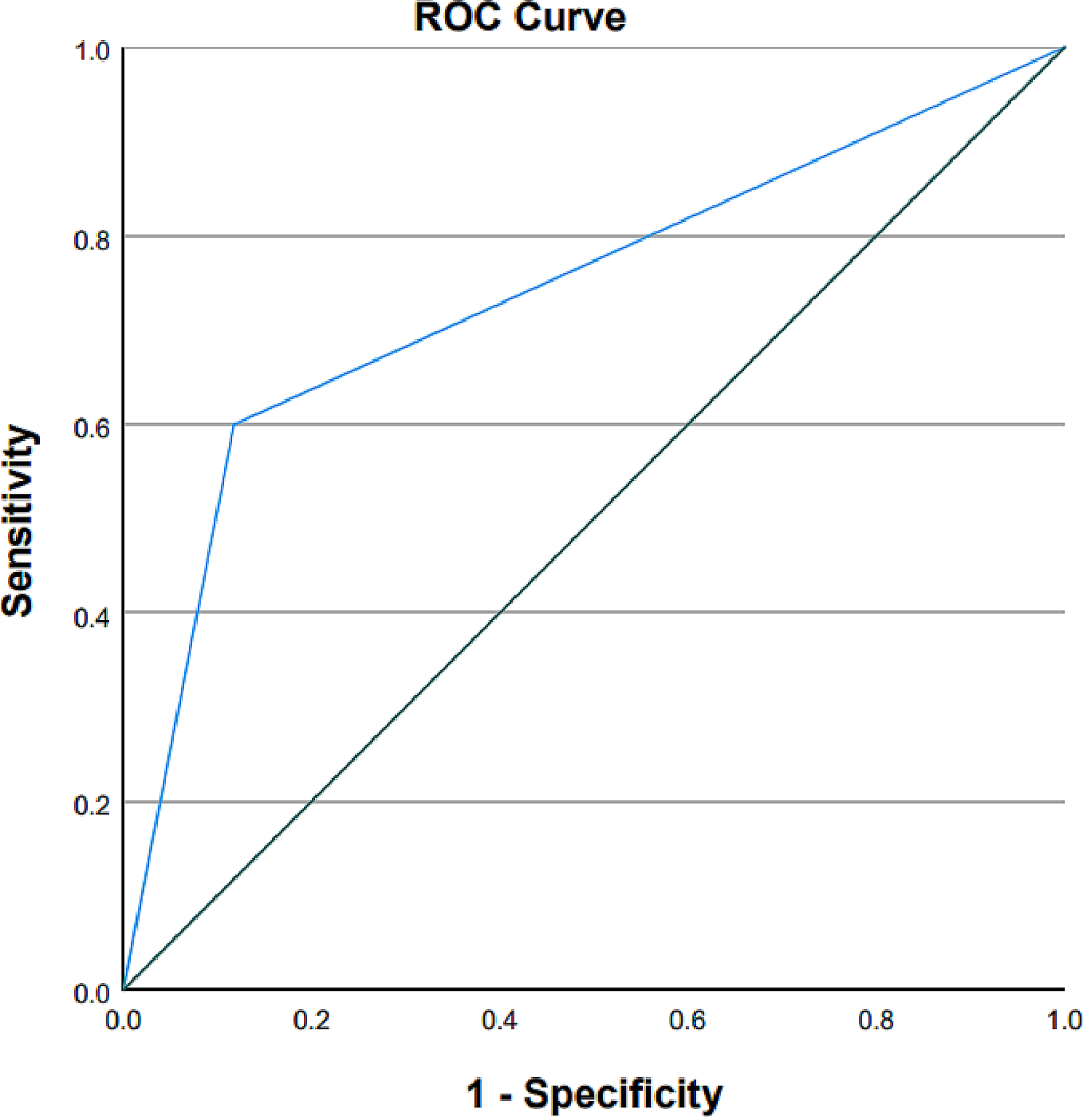
ROC curve for predictive value of post-treatment abortion for the occurrence of RCSP

The univariate analysis revealed that the RCSP group had significantly more pregnancies (median 5.0 vs. 4.0), higher abortion rates after treatment (60.0% vs. 11.8%), and lower live birth rates (13.3% vs. 48.2%) than the non-RCSP group (*P* < 0.05, Table 3). Meanwhile, no significant differences were observed in the other indicators (*P* > 0.05, Table 3). The multivariate logistic regression analysis showed that post-treatment abortion in patients with CSP was a risk factor for RCSP (odds ratio [OR] 11.25, 95% confidence interval [CI], 3.302–38.325; *P* < 0.01, Table 4). An ROC curve (Fig. 2) was drawn to evaluate the predictive value of post-treatment abortion for the occurrence of RCSP, and the AUC was calculated. The results revealed an AUC of 0.741, suggesting that abortion after treatment has a predictive value for the occurrence of RCSP.

**Table 3:** Comparison of clinical data for pregnancy outcomes in RCSP and non-RCSP groups

Clinical Coefficients	RCSP group (*n* = 15)	Non-RCSP group (*n* = 85)	t / χ2 /z	*P*
Age [years, M (P-25P75)]	33.0 (29.0–35.0)	32.0 (30.0–36.0)	0.532	0.940
Length of stay [days, M (P25-P75)]	5.0 (4.0–6.0)	5.0 (4.0–6.0)	0.350	1.000
Pregnancy [times, M (P25-P75)]	5.0 (5.0–7.0)	4.0 (3.0–5.0)	1.554	0.016
Yield [times, M (P25-P75)]	1.0 (1.0-1.3)	1.0 (1.0–1.0)	0.238	1.000
Cesarean section[times, M (P25-P75)]	1.0 (1.0–1.0)	1.0 (1.0–1.0)	0.238	1.000
Abortion [times, M (P25-P75)]	3.0 (2.0–5.0)	2.0 (1.0–3.0)	1.246	0.090
Weeks of menopause [weeks, M (P25-P75)]	7.0(6.2-9.0)	7.10 (6.2–8.3)	0.394	0.998
Gestational sac diameter [cm, M(P25-P75)]	2.60(1.7–4.8)	2.7 (2.1–3.9)	0.532	0.940
MT [cm, M (P25-P75)]	0.3(0.2–0.4)	0.3(0.2–0.4)	0.672	0.757
BMI [kg/m2, M (P25-P75)]	20.0 (18.8–22.2)	21.6 (20.2–24.1)	1.232	0.096
Method of surgery				
Aterine aspiration [n(%)]	73.3% (11/15)	80.0% (68/85)		
Laparoscopic surgery [n(%)]	26.7% (4/15)	20.0% (17/85)	1.748	0.417
UAE [n(%)]	26.7% (4/15)	16.5% (14/85)	0.898	0.343
Major bleeding [n(%)]	6.7% (1/15)	1.2% (1/85)	1.961	0.161
Live birth [n(%)]	13.3% (2/15)	48.2% (41/85)	6.337	0.012
Abortion after treatment [n(%)]	60.0% (9/15)	11.8% (10/85)	19.275	< 0.01
IUA [n(%)]	6.7% (1/15)	11.8% (10/85)	0.338	0.561
PCSP [n(%)]	6.7% (1/15)	8.2% (7/85)	0.043	0.836
Secondary infertility [n (%)]	26.7% (4/15)	40.0% (34/85)	0.962	0.327
Type of CSP				
I [n(%)]	40.0% (6/15)	40.0% (34/85)		
II [n(%)]	53.3% (8/15)	28.2% (24/85)	1.025	0.599
III [n(%)]	6.7% (1/15)	31.8% (27/85)		

CSP: Caesarean scar pregnancy; RCSP: Recurrent caesarean scar pregnancy; UAE: Artery embolization; MT: Myometrial thickness; IUA: intrauterine adhesions; PCSP: persistent caesarean scar pregnancy

**Table 4:** Independent risk factors for RCSP by multivariable logistic regression analysis

Parameter	B	SE	OR(95%CI)	*P*
Abortion after treatment	2.350	0.642	11.25(3.302,38.325)	<0.01

OR were calculated by logistic regression analysis with adjustments of number of Pregnancies, live birth rate and abortions after treatment, OR = odds ratio, B = Regression coefficient; SE = standard error;95% CI = 95% confdence interval

### Analysis of influencing factors in patients with CSP with secondary infertility outcomes

The univariate analysis revealed that patients in the secondary infertility group had significantly lower live birth (0% vs. 69.4%) and abortion rates after treatment (0% vs. 30.6%) and a higher incidence of IUA (26.3% vs. 1.6%) than the non-secondary infertility group (*P* < 0.05, Table 5). The other indicators were not statistically significant (*P* > 0.05; Table 5). The results of the univariate analysis were incorporated into the multivariate analysis, and none were influential factors for secondary infertility after CSP treatment.

**Table 5:** Comparison of clinical data for pregnancy outcomes in secondary infertility and non-secondary infertility groups

Clinical Coefficients	Infertility group (*n* = 38)	Non-infertility infertile group (*n* = 62)	t / χ2 /z value	*P*
Age [years, M (P-25P75)]	34.0 (30.8–37.3)	31.0 (29.0–34.0)	1.281	0.075
Length of stay [days, M (P25-P75)]	5.0 (4.0–7.0)	5.0 (4.0–5.0)	0.898	0.395
Gestation [times, M (P25-P75)]	4.5 (3.0–6.0)	4.0 (3.0–5.0)	0.552	0.921
Yield [times, M (P25-P75)]	1.0 (1.0-1.3)	1.0 (1.0–1.0)	0.445	0.989
Cesarean section [times, M (P25-P75)]	1.0 (1.0–1.0)	1.0 (1.0–1.0)	0.474	0.978
Abortion [times, M (P25-P75)]	2.0 (1.0–3.3)	2.0 (1.0–3.0)	0.375	0.999
Weeks of menopause [days, M (P25-P75)]	7.45 (6.50–9.33)	6.60 (6.18–7.63)	1.080	0.194
Gestational sac diameter [cm, M(P25-P75)]	3.25 (2.15-5.00)	2.60 (2.00-3.60)	1.372	0.059
MT [cm, M (P25-P75)]	0.24 (0.19–0.40)	0.32 (0.21–0.40)	1.088	0.187
BMI [kg/m2, *M* (P25-P75)]	21.61 (20.20-23.61)	21.27 (19.73–24.04)	0.738	0.648
**Mode of surgery**				
Aterine aspiration [n(%)]	68.4% (26/38)	85.5% (53/62)		
Laparoscopic surgery [n(%)]	31.6% (12/38)	14.5% (9/62)	4.242	0.120
UAE[n(%)]	21.1% (8/38)	16.1% (10/62)	0.387	0.534
Major bleeding [n(%)]	5.3% (2/38)	0% (0/62)	3.330	0.068
Live birth [n(%)]	0% (0/38)	69.4% (43/62)	46.237	< 0.01
Abortion after treatment [n(%)]	0% (0/38)	30.6% (19/62)	14.377	< 0.01
IUA[n(%)]	26.3% (10/38)	1.6% (1/62)	14.685	< 0.01
PCSP [n(%)]	7.9% (3/38)	8.1% (5/62)	0.001	0.976
RCSP [n (%)]	10.5% (4/38)	17.7% (11/62)	0.962	0.327
**Incision of CSP**				
I [n(%)]	34.2% (13/38)	54.8% (34/62)		
II [n(%)]	47.4% (18/38)	38.7% (24/62)	5.622	0.060
III [n(%)]	18.4% (7/38)	6.5% (4/62)		

CSP: Caesarean scar pregnancy; RCSP: Recurrent caesarean scar pregnancy; UAE: Artery embolization; MT: Myometrial thickness; IUA: intrauterine adhesions; PCSP: persistent caesarean scar pregnancy

## Discussion

We conducted a retrospective study, including as many samples as possible, with a 5-year follow-up period to identify the factors influencing reproductive outcomes in patients with CSP after treatment. Our study showed that the majority (73.6%) of women did not want to become pregnant again after CSP treatment, mainly because of concerns regarding RCSP and its complications [16, 17]. Patients with CSP have experienced three RCSPs [18], and some women even experienced five consecutive CSPs [19]. Four of our patients experienced three CSPs, and these four women also withdrew their subsequent intention to have children because of fear of RCSP. The cause of RCSP remains unclear. However, in our study, the incidence of RCSP was only 15%, which was significantly lower than the 43% incidence of live births; and placental implantation accounted for only 2.3% of live births. Therefore, the incidence of RCSP is low. These results are consistent with those reported by Nagi et al. [20]. We believe that the low risk of recurrence suggests that a CSP is more likely to be an incidental event than one caused by GS implantation into a scar site with greater affinity. Moreover, we do not support the idea [21] that the uterine scar is repaired during or after ectopic pregnancy of the CS scar to reduce the risk of recurrence. Therefore, most patients with CSP and fertility intentions should be encouraged to become pregnant. Very few pregnant patients with incisions require laparoscopic surgical repair.

Sadeghi et al. [22] have reported that 59 women with CSP became pregnant again, and the incidence of RCSP was 25%. Wang et al. [23] have reported that the incidence of RCSP was 15.6% during the fertility follow-up of 189 patients with CSP. Currently, few reports on RCSP with large sample sizes are available. Several studies with small sample sizes have demonstrated that a history of multiple miscarriages, multiple CS histories, poor medical resources, gestational age at the time of previous CSP treatment, and treatment methods are possible risk factors for RCSP [24–27]. Some scholars believe that the risk of RCSP may be directly related to the size of the anterior uterine wall defect and that laparoscopic resection of the lesion while repairing the scar during CS can reduce the risk of RSCP [28, 29]. However, studies have also suggested that the repair of scar defects cannot reduce the risk of RCSP and may lead to complications such as poor scar healing and pelvic adhesion; therefore, it is only applicable to patients with specific conditions, such as evident exudation of the GS and abnormally large uterine scar defects [30]. This study determined that abortion after surgical treatment was the only risk factor of RCSP occurrence. We believe that this may be attributable to abortion damaging the basal layer of the endometrium caused by CSs, resulting in the loss of glandular epithelial cells in the basal layer of the endometrium, an inability to repair the functional layer of the endometrium, and loss of re-epithelialisation in the scar area of the incision. Hence, repeated abortions can damage the endometrium or muscle layer, and damage to the integrity of the anterior wall of the uterus will lead to poor scar healing, resulting in the absence of decidua in the endometrial stroma, thus creating conditions for the reimplantation of fertilised eggs in the scar [31–34], and ultimately the development of RCSP. This also reminds us that if abortion is unavoidable in patients with CSP, selecting the treatment method with the least damage to the endometrium to terminate pregnancy as far as possible is necessary.

Currently, most studies on infertility in patients with CSP after treatment are based on medical records. A previous study has reported that the total incidence of infertility in patients with CSP after conservative and surgical treatment is 15.7% (8/51) [35]. Jurkovic et al. have reported that the incidence of secondary infertility after ultrasound-guided uterine aspiration in 79 patients with CSP was 16.5% [36]. Tang et al. have identified that the incidence of secondary infertility in women treated with hysteroscopy for CSP was 40% [37]. Chen et al. have reported that the incidence of secondary infertility after UAE combined with high-intensity focused ultrasound or UAE combined with uterine clearance was 23.7% in 135 patients with CSP [38]. The inconsistent incidence of infertility reported in the literature may be related to the length of follow-up. In this study, the incidence of infertility was 38%, and some patients were followed up for < 2 years. This probability continues to decline over time.

### Limitations

Different surgical methods did not affect pregnancy outcomes after CSP treatment. However, we included only two treatment modalities: ultrasound-guided uterine aspiration and laparoscopic gestational resection plus scar repair. We did not include patients who received other treatment modalities, such as conservative treatment, transvaginal surgery, hysteroscopic surgery, or high-intensity focused ultrasound. This aspect should be addressed in future studies. In addition, a larger sample size, longer follow-up duration, and a multicentre, prospective, randomised, double-blind research scheme will reduce experimental bias and provide better results in the future.

## Conclusions

The reproductive outcomes of women after CSP treatment were favourable, and no significant difference in reproductive outcomes was observed between women treated with uterine aspiration and those who underwent laparoscopy. Women who miss treatment for CSP should be vigilant regarding the risk of RCSP. All women in this study who became pregnant again were closely monitored in the first trimester, and those with RCSP were treated for pregnancy termination in the first trimester. Only one woman who had a live birth experienced placental accretion with major bleeding. None of the women had uterine ruptures or undergone hysterectomy. Therefore, if sufficient attention is paid to early diagnosis using imaging, serious CSP complications can be avoided.

## Data Availability

The data that support the findings of this study are available on request from the corresponding author Yan Lei upon reasonable request.

## Abbreviations

AUC: area under the curve
BMI: body mass index
CI: confidence interval
CS: Caesarean section
CSP: Caesarean scar pregnancy
GS: gestational sac
IUA: intrauterine adhesion
MT: myometrial thickness
OR: odds ratio
PCSP: persistent Caesarean scar pregnancy
RCSP: recurrent Caesarean scar pregnancy
ROC: receiver operating characteristic
UAE: uterine artery embolisation
